# Ferulic acid inhibits ox-LDL-induced ferroptosis and apoptosis in RAW 264.7 cells via the HIF-1 signaling pathway

**DOI:** 10.3389/fphar.2025.1524736

**Published:** 2025-03-18

**Authors:** Xize Wu, Xue Pan, Jian Kang, Yuxi Huang, Jiaqi Ren, Jiaxiang Pan, Kaifeng Yu, Yue Li

**Affiliations:** ^1^ The First Clinical College, Liaoning University of Traditional Chinese Medicine, Shenyang, China; ^2^ College of Traditional Chinese Medicine, Dazhou Vocational College of Chinese Medicine, Dazhou, Sichuan, China; ^3^ Department of Cardiology, Affiliated Hospital of Liaoning University of Traditional Chinese Medicine, Shenyang, China

**Keywords:** the HIF-1 signaling pathway, mitochondrion, lipid metabolism, cell death, ferulic acid

## Abstract

**Objective:**

Ferulic acid (FA) has shown potential in treating atherosclerosis (AS) by improving lipid metabolism and exerting anti-hypoxic effects. This study aimed to validate the mechanism of FA in AS through *in vitro* experiments.

**Methods:**

Network analysis was employed to predict the mechanisms underlying the therapeutic effects of FA on AS. An *in vitro* foam cell model was established using RAW 264.7 cells treated with ox-LDL. Cellular lipid accumulation was detected using Oil Red O staining; cell viability was assessed by cell counting kit-8; mitochondrial morphology and function were evaluated by transmission electron microscopy and JC-1 staining; apoptosis levels were detected by TUNEL and DAPI staining; mitochondrial Fe^2+^ content was measured by Mito-FerroGreen; and Western blot was performed to determine the protein expression levels of HIF-1α, Bax, Bcl2, GPX4, and EGFR.

**Results:**

Network analysis suggested that FA may exert its therapeutic effects on AS through the HIF-1 signaling pathway and is closely associated with the regulation of ferroptosis and apoptosis. FA upregulated the expression of ALOX5, BCL2, ERN1, GPX4, NOS3, and SLC2A1 mRNA and downregulated the expression of BAX, CYCS, EGFR, FLT1, HIF1A, NFKB1, NOS2, PARP1, and STAT3 mRNA. *In vitro* experiments demonstrated that FA reduces lipid accumulation, increases cell viability, improves mitochondrial function, and decreases reactive oxygen species content. Additionally, FA inhibited ferroptosis and apoptosis by suppressing the HIF-1 signaling pathway, up-regulating the expression of GPX4 and Bcl2, and down-regulating the expression of HIF-1α and Bax protein. HIF-1 agonists reversed these effects by activating the HIF-1 signaling pathway.

**Conclusion:**

FA improves mitochondrial function and suppresses ferroptosis and apoptosis by inhibiting the HIF-1 signaling pathway, thereby treating AS.

## 1 Introduction

Atherosclerosis (AS) is a chronic inflammatory condition that manifests as pathological changes within the vascular walls, hallmarked by lipid deposition and immune cell infiltration. It is also the pathological basis of various cardiovascular diseases, which are the primary cause of disease mortality in China and even the world, and are gradually increasing (Fan and Watanabe, 2022; Ma et al., 2020). The treatment of AS includes lifestyle interventions, lipid modulation, antiplatelet, and anti-inflammatory drugs, with lipid-lowering therapies occupying a central place (Libby, 2021). Timely enhancement of lipid metabolism, implementation of lipid-lowering therapy, and mitigation of risk factors for atherosclerotic cardiovascular diseases can effectively control the progression and poor prognosis associated with cardiovascular diseases. Numerous clinical studies have demonstrated that intensive, long-term lipid-lowering therapy not only slows the progression of AS but may also induce regression of atherosclerotic lesions (Kim et al., 2022; Nissen et al., 2006). Consequently, proactive lipid-lowering therapy is crucial for the prevention and management of cardiovascular diseases.

Ferroptosis is a unique form of regulated cell death, primarily characterized by intracellular iron ion accumulation and overproduction of lipid peroxides. Disturbances in iron metabolism (e.g., iron overload) and antioxidant system dysfunction (e.g., GPX4) are central to ferroptosis (Jiang et al., 2021). Studies have shown that ferroptosis induces endothelial cell dysfunction, significantly increasing vascular permeability and promoting the release of inflammatory factors, thereby accelerating the formation of atherosclerotic plaques. Additionally, ferroptosis leads to lipid peroxidation in macrophages, further promoting the formation of foam cells and exacerbating the accumulation of the lipid core within plaques. Meanwhile, cell death and inflammatory responses triggered by ferroptosis significantly increase the risk of plaque rupture, potentially leading to the occurrence of acute cardiovascular events (Jiang et al., 2021; Ouyang et al., 2021; Fang et al., 2024). In addition to ferroptosis, apoptosis, a genetically controlled cell death process, also plays a significant role in AS development, where it mainly affects endothelial cells, smooth muscle cells, and macrophages. Endothelial cell apoptosis is considered a pivotal early event in AS, leading to impaired vascular barrier function, promoting monocyte infiltration and inflammatory responses. Smooth muscle cell apoptosis weakens the structural integrity of the vascular wall and increases plaque instability. Moreover, apoptotic macrophages are not efficiently removed and form a necrotic core that further promotes plaque progression and increases the risk of plaque rupture and thrombosis (Duan et al., 2021; Clarke et al., 2006). Therefore, inhibition of ferroptosis and apoptosis is important for the treatment of AS, contributing to plaque stabilization, improvement of vascular function, and reduction of cardiovascular events.

Ferulic acid (FA, 3-methoxy-4-hydroxycinnamic acid, CAS: 1,135-24–6) is a phenolic substance recognized as a significant metabolite in numerous herbals. It has exhibited diverse bioactivities, including antioxidant, anti-inflammatory, endothelial protective, antifibrotic, antiapoptotic, hypoxia-protective, and antiplatelet aggregation effects. FA and its derivatives, such as piperazine ferulate tablets and sodium ferulate, have been approved by the Food and Drug Administration for treating metabolic and cardiovascular diseases (Li et al., 2021). Numerous randomized controlled trials have demonstrated the efficacy of FA and its derivatives in treating coronary atherosclerotic heart disease by promoting blood circulation, eliminating blood stasis, enhancing hemodynamics and vascular function, and ameliorating myocardial ischemia, thereby slowing the progression of AS (Rodriguez-Mateos et al., 2016; Shen et al., 2023). Additionally, they improve lipid profiles, reduce oxidative stress, inhibit the production of oxidized low-density lipoprotein (ox-LDL), and mitigate inflammatory responses in hyperlipidemia patients (Berger et al., 2005; Bumrungpert et al., 2018). Although FA has shown efficacy in treating AS and its mechanisms have been progressively elucidated, its role in inhibiting ferroptosis and apoptosis has rarely been explored. This study utilizes *in vitro* experiments to investigate whether FA regulates ferroptosis and apoptosis through the HIF-1 signaling pathway to treat AS, offering a deeper understanding of FA’s mechanisms in treating AS.

## 2 Materials and methods

### 2.1 Network analysis

#### 2.1.1 FA and disease-related target acquisition

The structure and canonical SMILES (COC1 = C(C=CC(=C1)C=CC(=O)O)O) of FA were imported into the Swiss TargetPrediction database (http://www.swisstargetprediction.ch/) for “probability”>0, the STITCH database (http://stitch.embl.de/) for “medium confidence (0.400)”, the SuperPred database (https://prediction.charite.de/index.php) for “probability” >50%, and the TCMSP database (https://www.tcmsp-e.com/) to predict FA potential targets.

AS-related genes were collected from the GeneCards database (https://www.genecards.org/) with a “relevance score” greater than the mean value (1.325) and from the OMIM database (https://omim.org/) using the keyword “atherosclerosis.”

Ferroptosis-related genes were obtained from the FerrDb database (http://www.zhounan.org/ferrdb/current/), and apoptosis-related genes were retrieved from the GeneCards database, screening for genes with a “relevance score” greater than 5.

#### 2.1.2 Functional enrichment analysis

The intersection targets were imported into the David database (https://david.abcc.ncifcrf.gov/) for functional enrichment analysis, with FDR<0.01 set as the screening condition.

#### 2.1.3 Molecular docking

The 3D structure of FA was obtained from the PubChem database (https://pubchem.ncbi.nlm.nih.gov/). The crystal structure of the protein was obtained from the PDB database (https://www.rcsb.org/). The protein receptor and small molecule ligand were dehydrated and hydrogenated using PyMOL software. The Molecular docking analysis was then performed using AutoDockTools, and the results were visualized using PyMOL software (Rosignoli and Paiardini, 2022; Morris et al., 2008). The binding energy was calculated, with lower values indicating better and more stable binding interactions.

### 2.2 Experimental validation

#### 2.2.1 Drugs and reagents

Reagents: FA (Solarbio, Cat.F8330); Dimethyl Sulfoxide (DMSO) (Solarbio, Cat.D8370); ox-LDL (Yiyuan, Cat.YB-002-1); Fetal Bovine Serum (Biosharp, Cat.BL205 A); Trypsin-EDTA Solution (Biosharp, Cat.BL512 A); Dulbecco’s Modified Eagle Medium (Pricella, Cat.PM150210); Penicillin/Streptomycin (Biosharp, Cat.BL505 A); Oil Red O (ORO) Dye Solution (Solarbio, Cat.G1262); Serum-Free Cell Freezing Medlum (Biosharp, Cat.BL203B); Paraformaldehyde, 4% (Solarbio, Cat.P1110); Cell Counting Kit-8 (Solarbio, Cat.CA1210); Reactive Oxygen Species (ROS) Assay Kit (BestBio, Cat. BB-47053); TUNEL (Beyotime, Cat.C1086); Mito-FerroGreen (DOJINDO, Cat.M489); Total Cholesterol Assay Kit (Abbkine, Cat.KTB2220); Free Cholesterol Assay Kit (Abbkine, Cat.ATTOC3001); Mounting Medium, Antifading (with DAPI) (Solarbio, Cat.S2110); JC-1 Dye (Beyotime, Lot.C2003S); Oltipraz (an inhibitor of the HIF-1 signaling pathway) (APExBIO, Lot.No.B59582133A132); FG-4592 (an agonist of the HIF-1 signaling pathway) (APExBIO, Lot.No.A418731337769); RNA Extraction Solution (Servicebio, Cat.G3013); Phosphate-Buffered Saline (PBS) (Servicebio, Cat.G4202); Chloroform Substitute (Servicebio, Cat.G3014); Chloroform Substitute (Servicebio, Cat.G3014); RNA Lysate (Servicebio, Cat.G3029); Water Nuclease-Free (Servicebio, Cat.G4700); SweScript All-in-One RT SuperMix for qPCR (One-Step gDNA Remover) (Servicebio, Cat.G3337); 2×Universal Blue SYBR Green qPCR Master Mix (Servicebio, Cat.G3326); Isopropanol (Sinopharm, Cat.80109218); Anhydrous Ethanol (Sinopharm, Cat.10009218); RIPA Lysis Buffer (Beyotime, Cat.P0013B); anti-HIF-1α (Affinity, Cat.BF8002); anti-EGFR (Proteintech, Cat.51071-2-AP); anti-GPX4 (Proteintech, Cat.67763-1-Ig); anti-Bcl2 (Abcam, Cat.ab32124); anti-Bax (Abcam, Cat.ab32503); β-Actin Rabbit mAb (Abclonal, Lot. AC038).

Instruments: Transmission Electron Microscope (TEM) (HITACHI, H-7650); Electronic Analytical Balance (METTLER TOLEDO, MS105DU); Inverted Fluorescence Microscope (Nikon, Eclipse Ci); Vortex Mixer (Servicebio, SMV-3500); Sealing Instrument (Servicebio, FS-A20); Microplate Reader (Tecan, Spark 10M); Microplate Centrifuge (Servicebio, SMP-2); High-Speed Frozen Microcentrifuge (DragonLab, D3024R); Fluorescent Quantitative PCR Instrument (Bio-rad, CFX Connect); PCR Instrument (Eastwin, ETC811).

Preparation of FA solution: 1.942 mg of FA powder was dissolved in 2 mL of DMSO (<0.1%) to prepare a 5 mM FA stock solution. Subsequently, 400 μL, 200 μL, 100 μL, and 50 μL of the stock solution were diluted with culture medium to a final volume of 1 mL to obtain 2.0 mM, 1.0 mM, 0.5 mM, and 0.25 mM FA solutions, respectively.

#### 2.2.2 Cell culture and grouping

RAW 264.7 cells were purchased from the iCell Bioscience Inc. (Shanghai) Co., Ltd. (Cat.CL-0190) and cultured in Dulbecco’s Modified Eagle Medium supplemented with 10% fetal bovine serum and 1% penicillin/streptomycin and incubated at 37°C in humidified 5% CO2.

Cells were divided into seven groups: ①Control group (CTRL): cells were cultured normally without any treatment; ②Mod group (MOD): cells were treated with ox-LDL (100 μg/mL) for 24 h; ③FA group (FA): cells were treated with ox-LDL and FA (1.0 mmol/L) for 24 h; ④FA and FG-4592 group (FA + FG-4592): cells were treated with ox-LDL, FA, and FG-4592 (an agonist of the HIF-1 signaling pathway) (400 μM) for 24 h; ⑤FG-4592 group (FG-4592): cells were treated with ox-LDL and FG-4592 for 24 h; ⑥FA and Oltipraz group (FA + Oltipraz): cells were treated with ox-LDL, FA, and Oltipraz (an inhibitor of the HIF-1 signaling pathway) (10 μM) for 24 h; ⑦Oltipraz group (Oltipraz): cells were treated with ox-LDL and Oltipraz for 24 h.

#### 2.2.3 ORO staining

RAW 264.7 cells were seeded in 6-well plates at a density of 2.0 × 10^5^ cells/mL (2 mL/well) and cultured for 24 h until adherence. After intervention, cells were fixed with 1 mL of 4% paraformaldehyde for 30 min at room temperature, followed by twice washes with PBS (5 min each). Cells were then stained with 1 mL of ORO solution (prepared by mixing ORO stock solution with distilled water at a 3:2 ratio and filtering) for 30 min at room temperature in the dark. After staining, cells were rinsed with 60% isopropanol for 1 min and washed twice with distilled water (5 min each). Lipid droplet formation was observed and photographed under an optical microscope.

#### 2.2.4 Determination of cholesteryl ester content

RAW 264.7 cells were seeded in 6-well plates at a density of 2.0 × 10^5^ cells/mL (2 mL/well) and cultured for 24 h until adherence. After intervention, cells were washed twice with cold PBS and lysed with 500 µL RIPA buffer on ice for 30 min. Lysates were centrifuged at 12,000 rpm for 10 min at 4°C, and 100 µL of supernatant was mixed with 300 µL isopropanol, vortexed, and incubated at room temperature for 10 min. After centrifugation (12,000 rpm, 10 min, 4°C), the pellet was resuspended in assay buffer and incubated at 37°C for 30 min.

#### 2.2.5 Cell viability assay

RAW 264.7 cells were seeded in 96-well plates at a density of 5.0 × 10^4^ cells/mL (100 μL/well) and cultured for 24 h until adherence. After intervention, 10 μL of CCK-8 reagent was added to each well, and plates were incubated for 2 h at 37°C. Absorbance (OD) was measured at 450 nm using a microplate reader.

#### 2.2.6 Transmission Electron Microscope

RAW 264.7 cells were seeded in 6-well plates at a density of 2.0 × 10^5^ cells/mL (2 mL/well) and cultured for 24 h until adherence. After intervention, cells were fixed with 1 mL of 1% osmium tetroxide solution for 2 h at 4°C and then washed three times with PBS (10 min each). Dehydration was performed using a graded ethanol series (50%, 70%, 80%, 90%, 95%, and 100%) for 15 min each, followed by two 10-min rinses with acetone. The samples were embedded in epoxy resin, polymerized at 60°C for 48 h, sectioned into ultrathin slices (70–90 nm), stained with uranyl acetate and lead citrate, and observed under a TEM.

#### 2.2.7 JC-1 staining

RAW 264.7 cells were seeded in 6-well plates at a density of 2.0 × 10^5^ cells/mL (2 mL/well) and cultured for 24 h until adherence. After intervention, cells were incubated with 1 mL of JC-1 staining solution (10 μM, dissolved in DMSO and diluted to 10 μM according to the manufacturer’s instructions) at 37°C for 30 min in the dark. Cells were washed twice with PBS (5 min each), and mitochondrial membrane potential was observed under a fluorescence microscope.

#### 2.2.8 ROS assay

RAW 264.7 cells were seeded in 6-well plates at a density of 2.0 × 10^5^ cells/mL (2 mL/well) and cultured for 24 h until adherence. After intervention, cells were incubated with 1 mL of DCFH-DA solution (10 μM, prepared by diluting 10 mM stock solution in serum-free medium at 1:1,000) at 37°C for 30 min in the dark. Cells were washed twice with PBS (5 min each), and intracellular ROS levels were observed under a fluorescence microscope.

#### 2.2.9 TUNEL staining

RAW 264.7 cells were seeded in 6-well plates at a density of 2.0 × 10^5^ cells/mL (2 mL/well) and cultured for 24 h until adherence. After intervention, cells were fixed with 4% paraformaldehyde for 20 min at room temperature and permeabilized with 0.1% Triton X-100 for 5–10 min. The TUNEL reaction mixture was prepared by mixing Terminal deoxynucleotidyl transferase (TdT) with FITC-conjugated dUTP according to the manufacturer’s instructions. The mixture was then added to the cell samples and incubated at 37°C for 1 h in the dark. After incubation, cells were washed three times with PBS (5 min each). Finally, apoptotic cells were observed under a fluorescence microscope.

#### 2.2.10 Fe^2+^ detection

RAW 264.7 cells were seeded in 6-well plates at a density of 2.0 × 10^5^ cells/mL (2 mL/well) and cultured for 24 h until adherence. After intervention, cells were incubated with 1 mL of Mito-FerroGreen working solution (5 μM, dissolved in DMSO and diluted to 5 μM according to the manufacturer’s instructions) at 37°C for 30 min in the dark. Cells were washed twice with PBS (5 min each), and mitochondrial Fe^2+^ levels were observed under a fluorescence microscope.

#### 2.2.11 Quantitative real-time polymerase chain reaction (qRT-PCR)

RAW 264.7 cells were seeded in 6-well plates at a density of 1.0 × 10^6^ cells/mL and cultured for 24 h until adherence. After intervention, total RNA was extracted from cells using Trizol reagent according to the manufacturer’s instructions and treated with Turbo DNase to remove genomic DNA contamination. RNA was transcribed into cDNA using a reverse transcription kit. Gene-specific primers were designed using Primer Express v3.0 software and synthesized by Sangon Biotechnology (Shanghai) Co., Ltd. (https://www.sangon.com/) (Table 1). Real-time PCR was performed using SYBR Select Master Mix (Applied Biosystems). Relative gene expression levels were calculated using the ^ΔΔ^Ct method, with β-actin/GAPDH as the internal control (Gumireddy et al., 2014).

**TABLE 1:** A list of the primers used in the qRT-PCR.

Gene	Primer sequence (5′-3′)
β-actin	Forword: CAC​CCA​GCA​CAA​TGA​AGA​TCA​AGA​T
	Reverse: CCA​GTT​TTT​AAA​TCC​TGA​GTC​AAG​C
ALOX5^b^	Forword: CCT​ATG​CCT​CCC​TGT​GCT​TTC​C
	Reverse: ACC​TGG​TCG​CCC​TCG​TAG​TAG
BAX^c^	Forword: ACC​AAG​AAG​CTG​AGC​GAG​TGT​C
	Reverse: TGT​CCA​CGG​CGG​CAA​TCA​TC
BCL2^c^	Forword: CTG​TGG​ATG​ACT​GAG​TAC​CTG​AAC​C
	Reverse: CAG​AGA​CAG​CCA​GGA​GAA​ATC​AAA​C
CYCS^c^	Forword: CCA​AAT​CTC​CAC​GGT​CTG​TTC
	Reverse: ATC​AGG​GTA​TCC​TCT​CCC​CAG
EGFR^a^ ^,^ ^b^ ^,^ ^c^	Forword: GAC​AGC​ATA​GAC​GAC​ACC​TTC​CTC
	Reverse: CTG​GCT​TGG​ACA​CTG​GAG​ACT​G
ERN1^c^	Forword: ACA​CTG​CCT​GAG​ACC​TTG​TTG
	Reverse: GGA​GCC​CGT​CCT​CTT​GCT​A
FLT1^a^	Forword: CCA​CCT​CTC​TAT​CCG​CTG​G
	Reverse: ACC​AAT​GTG​CTA​ACC​GTC​TTA​TT
GPX4^b^	Forword: CGA​TAC​GCT​GAG​TGT​GGT​TTG​C
	Reverse: TGC​CCT​TGG​GTT​GGA​TCT​TCA​TC
HIF1A^a^	Forword: CAC​CGC​CAC​CAC​CAC​TGA​TG
	Reverse: TGA​GTA​CCA​CTG​TAT​GCT​GAT​GCC
NFKB1^a^	Forword: ATG​GCA​GAC​GAT​GAT​CCC​TAC
	Reverse: TGT​TGA​CAG​TGG​TAT​TTC​TGG​TG
NOS2^a^	Forword: GTT​CTC​AGC​CCA​ACA​ATA​CAA​GA
	Reverse: GTG​GAC​GGG​TCG​ATG​TCA​C
NOS3^a^	Forword: GGC​TGG​GTT​TAG​GGC​TGT​G
	Reverse: CTG​AGG​GTG​TCG​TAG​GTG​ATG
PARP1^c^	Forword: GGC​AGC​CTG​ATG​TTG​AGG​T
	Reverse: GCG​TAC​TCC​GCT​AAA​AAG​TCA​C
PIK3R1^a^ ^,^ ^c^	Forword: GTG​CAT​GGA​CTG​TTT​CCA​ATA​CA
	Reverse: AAT​GAC​GGA​CTT​CTC​ACT​TCA​C
RELA^a^ ^,^ ^c^	Forword: AGG​CTT​CTG​GGC​CTT​ATG​TG
	Reverse: TGC​TTC​TCT​CGC​CAG​GAA​TAC
SLC2A1^a^	Forword: GCT​TCT​CCA​ACT​GGA​CCT​CAA​A
	Reverse: GAA​GAA​CAG​AAC​CAG​GAG​CAC​AG
STAT3^a^	Forword: CAA​TAC​CAT​TGA​CCT​GCC​GAT
	Reverse: GAG​CGA​CTC​AAA​CTG​CCC​T
TLR4^a^ ^,^ ^b^ ^,^ ^c^	Forword: GTC​GGT​CCT​CAG​TGT​GCT​TGT​AG
	Reverse: CTC​ATT​CCT​TAC​CCA​GTC​CTC​ATC​C
GAPDH	Forword: GGA​CGC​TTT​CTT​TCC​TTT​CG
	Reverse: CAG​GCT​TTC​CTA​ACG​GCT​G
ND1	Forword: TCC​TAA​TGC​TTA​CCG​AAC​GAA​A
	Reverse: ATG​GTA​GAT​GTG​GCG​GGT​TT

Note:

^a^ The HIF-1 pathway-related genes.

^b^ ferroptosis-related genes.

^c^ apoptosis-related genes.

#### 2.2.12 Western blot

RAW 264.7 cells were seeded in 6-well plates at a density of 1.0 × 10^6^ cells/well and cultured for 24 h until adherence. After intervention, total protein extracts were obtained using RIPA Lysis Buffer supplemented with 1× protease inhibitor cocktail. Protein concentrations were determined using the Enhanced BCA Protein Assay Kit. Proteins were separated by SDS-PAGE and transferred to PVDF membranes. The membranes were blocked with 5% non-fat milk in TBST for 1 h at room temperature and then incubated overnight at 4°C with primary antibodies: anti-HIF-1α, anti-GPX4, anti-Bcl2, anti-Bax, and anti-EGFR. Membranes were washed with TBST and incubated with a secondary antibody conjugated to horseradish peroxidase for 2 h at room temperature. Protein bands were detected using the ECL Ultra method on a DNR bioimaging system. Band intensity was quantified using ImageJ software, and protein expression levels were normalized to β-actin.

### 2.3 Statistical analysis

Photographs were taken under fluorescence microscope observation and analyzed quantitatively using ImageJ. Results are expressed as mean ± standard deviation. Statistical analyses were performed using GraphPad Prism 9.5 for data analysis and visualization. One-way ANOVA and Tukey HSD *post hoc* tests were used when normality and homogeneity of variance were met; Kruskal–Wallis H tests were used when these not met. A *P* < 0.05 considered statistically significant.

## 3 Results

### 3.1 FA May ameliorate AS via the HIF-1 signaling pathway

**FIGURE 1:**
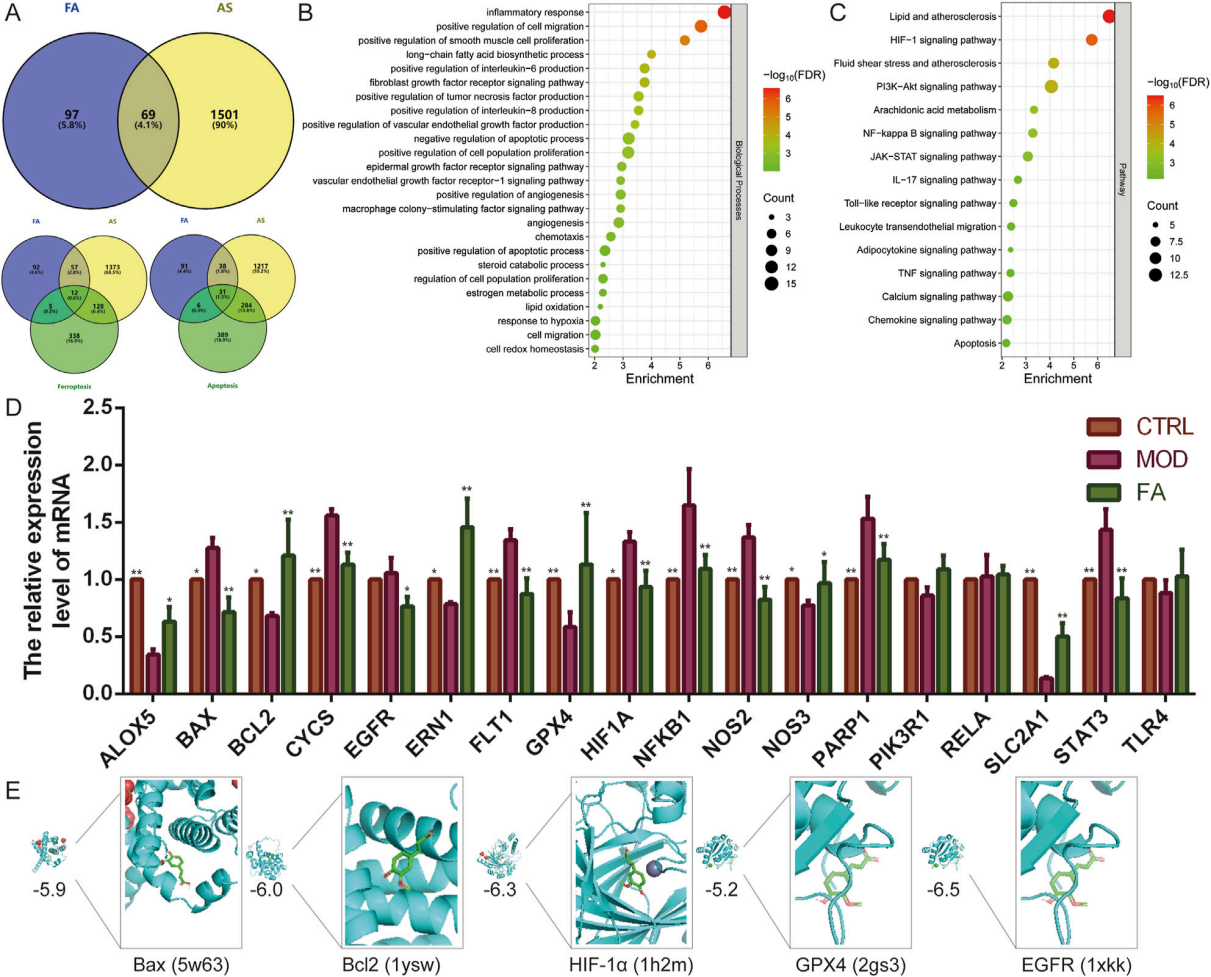
Network analysis predicts potential mechanisms for FA treatment of AS. **(A)** Venn diagram showing 69 intersection targets of FA for AS, of which 12 are related to ferroptosis and 31 to apoptosis. **(B–C)** The **(B)** biological processes and **(C)** pathways enrichment analysis of intersection targets. **(D)** qRT-PCR was performed to detect the effect of FA on the expression of genes related to the HIF-1 signaling pathway (EGFR, FLT1, HIF1A, NFKB1, NOS2, NOS3, PIK3R1, RELA, SLC2A1, STAT3, and TLR4), ferroptosis (ALOX5, EGFR, GPX4, and TLR4), and apoptosis (BAX, BCL2, CYCS, EGFR, ERN1, NFKB1, PARP1, PIK3R1, RELA, and TLR4) (*n* = 6). **(E)** Molecular docking of FA with Bax, Bcl2, HIF-1α, GPX4, and EGFR proteins. Results are expressed as the mean ± S.D. ^*^ *P* < 0.05, ^**^ *P* < 0.01 vs the MOD group.

A total of 166 predicted targets for FA, 1,570 diseases, 484 ferroptosis-related genes, and 710 apoptosis-related genes were retrieved from the database after eliminating overlaps. Subsequently, 69 intersection targets for FA treatment of AS were identified, including 12 related to ferroptosis and 31 related to apoptosis (Figure 1A).

Functional enrichment analysis of these 69 intersection targets revealed 62 biological processes and 70 signaling pathways (FDR < 0.01). These biological processes were associated with inflammatory responses (e.g., positive regulation of interleukin-6, interleukin-8, and tumor necrosis factor production), cell migration (e.g., positive regulation of smooth muscle cell proliferation and chemotaxis), apoptotic processes, hypoxia, angiogenesis (e.g., positive regulation of vascular endothelial growth factor production, angiogenesis, and vascular endothelial growth factor receptor-1 signaling pathway), immune response (e.g., macrophage colony-stimulating factor signaling pathway), and oxidative stress (e.g., lipid oxidation and cell redox homeostasis). Key signaling pathways involved included HIF-1, PI3K-Akt, NF-kappa B, JAK-STAT, and IL-17 (Figure 1B, C). Additionally, further enrichment analysis of the 12 ferroptosis-related targets and 31 apoptosis-related targets demonstrated that all were significantly enriched in the HIF-1 signaling pathway (FDR<0.01).

To further investigate the effects of FA, a foam cell model was constructed using 100 μg/mL ox-LDL-induced RAW 264.7 cells. Subsequently, qRT-PCR was used to examined the expression of genes related to the HIF-1 signaling pathway (EGFR, FLT1, HIF1A, NFKB1, NOS2, NOS3, PIK3R1, RELA, SLC2A1, STAT3, and TLR4), ferroptosis (ALOX5, EGFR, GPX4, and TLR4), and apoptosis (BAX, BCL2, CYCS, EGFR, ERN1, NFKB1, PARP1, PIK3R1, RELA, and TLR4). Compared to the CTRL group, the MOD group exhibited altered expression of 14 genes. Specifically, the expression of BAX (*P* < 0.05), CYCS (*P* < 0.01), FLT1 (*P* < 0.01), HIF1A (*P* < 0.05), NFKB1 (*P* < 0.01), NOS2 (*P* < 0.01), PARP1 (*P* < 0.01), and STAT3 (*P* < 0.01) was significantly upregulated (*P* < 0.05), while the expression of ALOX5 (*P* < 0.01), BCL2 (*P* < 0.05), ERN1 (*P* < 0.05), GPX4 (*P* < 0.01), NOS3 (*P* < 0.05), and SLC2A1 (*P* < 0.01) was significantly downregulated (*P* < 0.05). In contrast, the expression of EGFR, PIK3R1, RELA, and TLR4 did not differ significantly (*P* > 0.05). FA treatment improved the expression of most genes (*P* < 0.05), except for PIK3R1 (*P* > 0.05), RELA (*P* > 0.05), and TLR4 (*P* > 0.05), compared to the MOD group. These results suggest that FA targets are distributed across multiple pathways, which interact and regulate each other (Figure 1D). The HIF-1 signaling pathway may play a central role in the mechanism of FA treatment of AS by regulating ferroptosis and apoptosis.

To further validate these findings, molecular docking was performed to assess the affinity of FA for targets related to the HIF-1 signaling pathway (HIF1A, EGFR), ferroptosis (GPX4), and apoptosis (BCL2, BAX). The results indicated that all five proteins exhibited strong binding affinity for FA, with binding energies less than −5.0 kcal/mol (Figure 1E).

### 3.2 FA attenuates ox-LDL-induced lipid accumulation and enhances cell viability in RAW 264.7 cells

**FIGURE 2:**
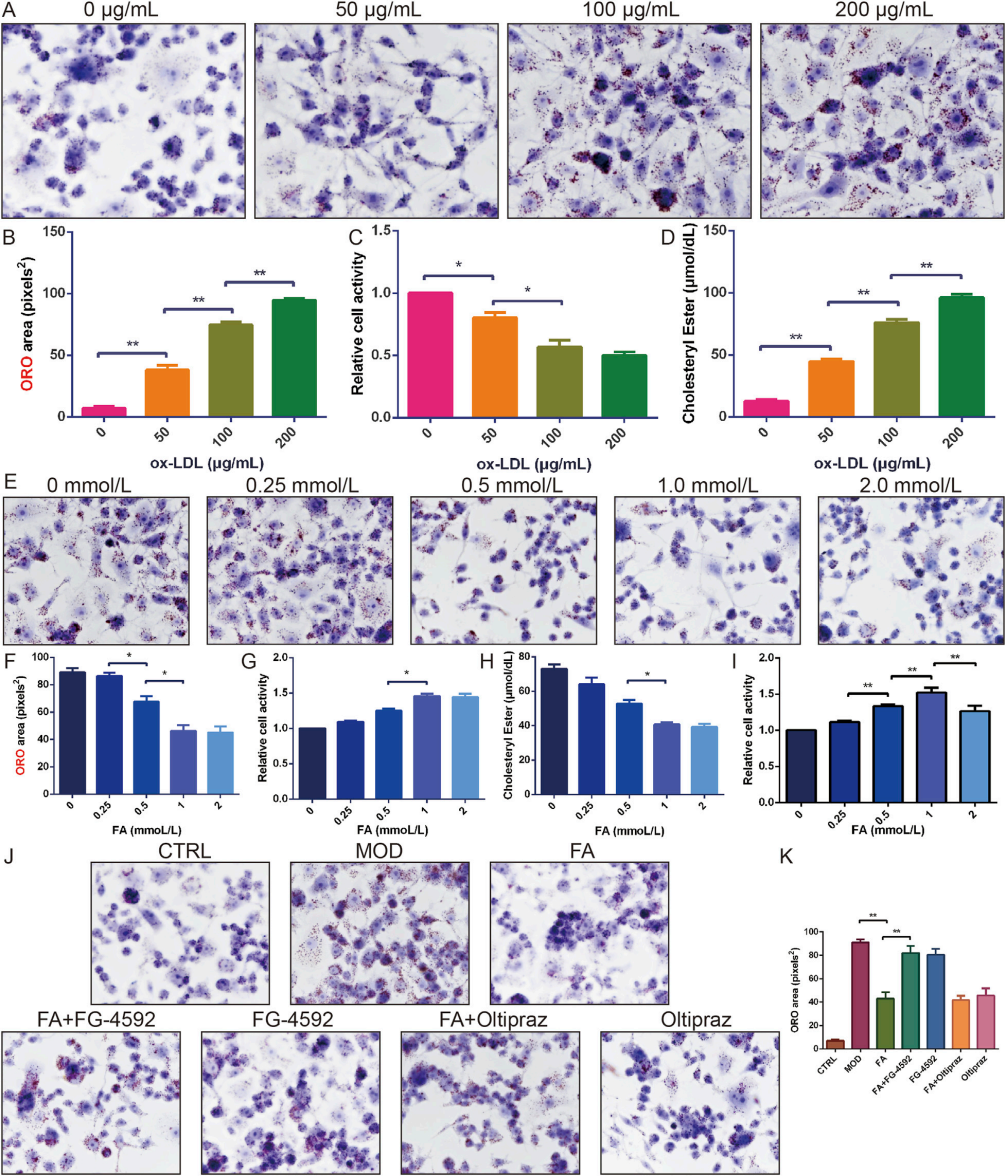
The effect of FA on ox-LDL-induced lipid accumulation and cell viability in RAW 264.7 cells. **(A-B)** ORO staining of RAW 264.7 cells treated with different doses of ox-LDL (*n* = 6, bar = 50 μm). **(C)** Relative cell viability of RAW 264.7 cells treated with different doses of ox-LDL (*n* = 10). **(D)** Cholesterol ester content of RAW 264.7 cells treated with different doses of ox-LDL (*n* = 6). **(E-F)** ORO staining of RAW 264.7 cells induced by ox-LDL and treated with different doses of FA (*n* = 6, bar = 50 μm). **(G)** Relative cell viability of RAW 264.7 cells induced by ox-LDL and treated with different doses of FA (*n* = 10). **(H)** Cholesterol ester content of RAW 264.7 cells induced by ox-LDL and treated with different doses of FA (*n* = 6). **(I)** Relative cell viability of RAW 264.7 cells treated with different doses of FA (*n* = 10). **(J-K)** ORO staining of RAW 264.7 cells induced by ox-LDL and treated with FA (*n* = 6, bar = 50 μm). Results are expressed as the mean ± S.D. ^*^ *P* < 0.05, ^**^ *P* < 0.01 vs the previous group.

To establish optimal modeling conditions, RAW 264.7 cells were treated with different concentrations of ox-LDL (0, 50, 100, and 200 μg/mL). Lipid accumulation and cell viability were assessed using ORO staining and the CCK-8 assay, respectively. Results showed that 100 μg/mL ox-LDL significantly increased lipid accumulation (*P* < 0.01) and cholesteryl ester content (*P* < 0.05), and reduced cell viability (*P* < 0.01) (Figures 2A–D). Thus, 100 μg/mL ox-LDL was selected for foam cell modelling.

Subsequently, the effects of different concentrations of FA (0, 0.25, 0.5, 1.0, and 2.0 mmol/L) on ox-LDL-induced lipid accumulation and cell viability were examined. FA treatment reduced cellular lipid accumulation and cholesteryl ester content in a concentration-dependent manner, with the most significant effect observed at 1.0 mmol/L without cytotoxicity (Figures 2E–H). However, 2.0 mmol/L FA decreased cell viability (*P* < 0.01) (Figure 2I). Thus, 1.0 mmol/L FA was selected for further mechanistic studies.

ORO staining revealed that the MOD, FA + FG-4592, and FG-4592 groups exhibited extensive cytoplasmic lipid deposition, accompanied by increased cell volume and altered morphology (*P* < 0.01). In contrast, foam cells treated with FA or Oltipraz significantly reduced lipid accumulation (*P* < 0.01), whereas FG-4592, a HIF-1α activator, antagonized the ameliorating effect of FA (*P* < 0.01) (Figure 2J, K), suggesting that FA attenuates ox-LDL-induced lipid accumulation and enhances cell viability in RAW 264.7 cells by inhibiting the HIF-1 signaling pathway.

### 3.3 FA improves ox-LDL-induced mitochondrial function and reduces ROS content in RAW 264.7 cells by inhibiting the HIF-1 signaling pathway

**FIGURE 3:**
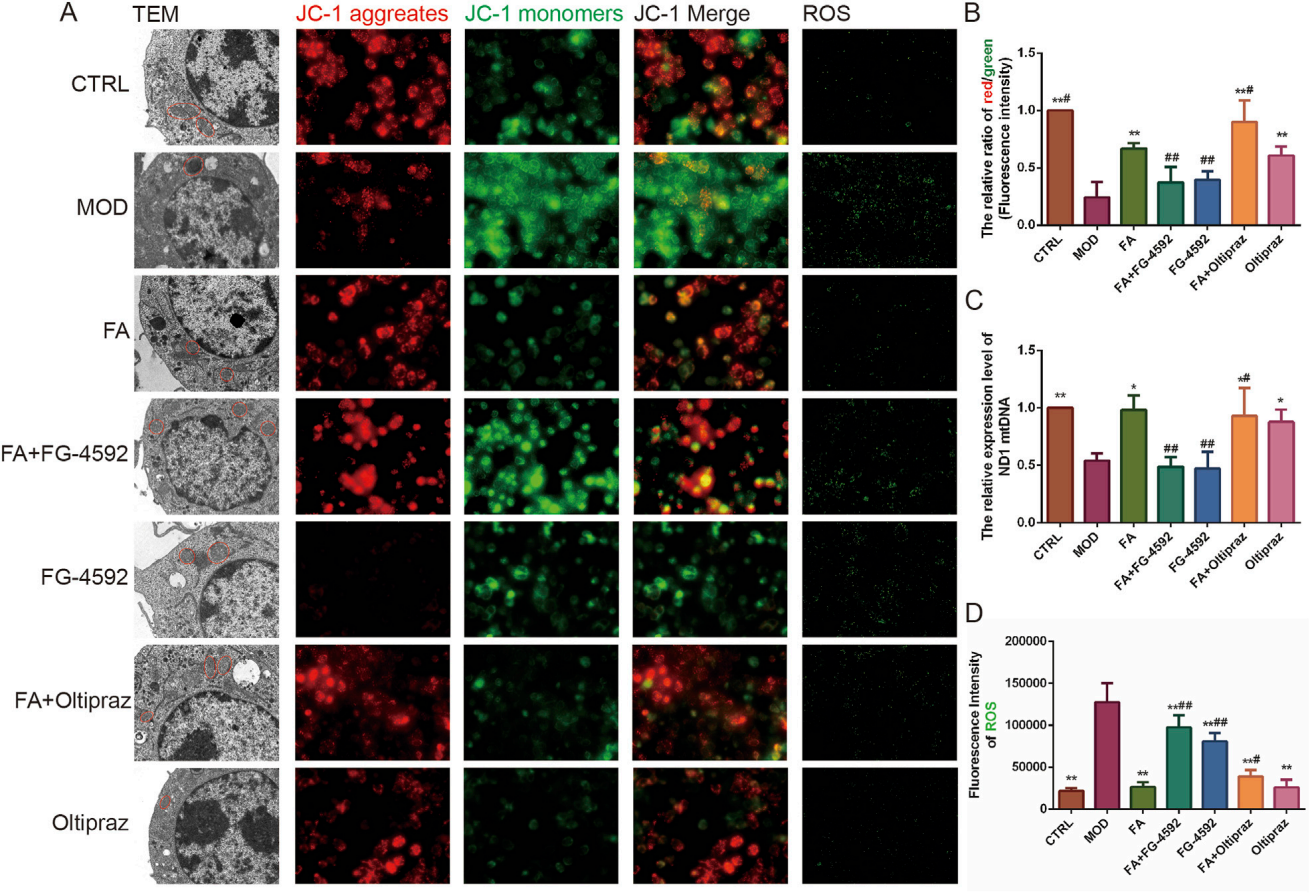
The effect of FA on ox-LDL-induced mitochondrial function in RAW 264.7 cells. **(A)** First column: mitochondrial morphology observed by transmission electron microscopy (bar = 1 μm); second to fourth columns: mitochondrial membrane potential detected by JC-1 staining (*n* = 15, bar = 500 μm); fifth column: ROS fluorescence detected by DCFH-DA (*n* = 15, bar = 500 μm). **(B)** Ratio of red/green fluorescence intensity in mitochondria. **(C)** Relative content of mitochondrial mtDNA (*n* = 6). **(D)** ROS contents detected by DCFH-DA. Results are expressed as the mean ± S.D. ^*^ *P* < 0.05, ^**^ *P* < 0.01 vs the MOD group; ^#^ *P* < 0.05, ^##^ *P* < 0.01. vs the FA group.

TEM revealed that mitochondria in the CTRL group exhibited intact structures with well-organized cristae, while those in the MOD, FG-4592, and FA + FG-4592 groups showed significant swelling and cristae disorganization. Treatment with FA, Oltipraz, or FA + Oltipraz effectively restored normal mitochondrial morphology (Figure 3A).

Mitochondrial function was assessed using JC-1 staining, which measures the mitochondrial membrane potential by the red/green fluorescence intensity ratio, where a higher ratio indicates better mitochondrial function. Additionally, mitochondrial DNA (mtDNA) levels were quantified using PCR. Both the fluorescence intensity ratio of mitochondrial membrane potential and mtDNA levels were significantly higher in the FA (*P* < 0.01) (*P* < 0.05), FA + Oltipraz (*P* < 0.01) (*P* < 0.05), and Oltipraz (*P* < 0.01) (*P* < 0.05) groups compared to the MOD group. However, FG-4592 antagonized the protective effect of FA (*P* < 0.01) (Figures 3A–C), suggesting that FA’s action is mediated through the HIF-1 signaling pathway.

ROS content, which reflects mitochondrial function, was measured and showed results consistent with JC-1 staining (Figure 3A, D). Collectively, these experiments demonstrate that FA improves mitochondrial function and reduces ROS content in ox-LDL-induced RAW 264.7 cells by inhibiting the HIF-1 signaling pathway.

### 3.4 FA inhibits ox-LDL-induced ferroptosis and apoptosis in RAW 264.7 cells by inhibiting the HIF-1 signaling pathway

**FIGURE 4:**
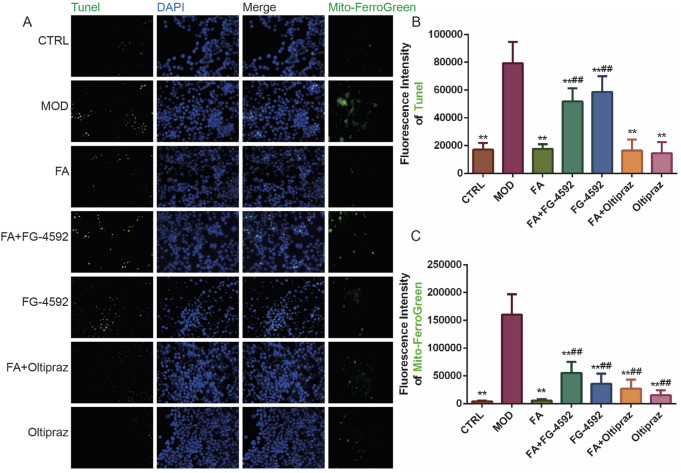
The effect of FA on ox-LDL-induced ferroptosis and apoptosis in RAW 264.7 cells. **(A)** First to three columns: apoptotic fluorescence detected by TUNEL and DAPI staining (bar = 500 μm); fourth column: mitochondrial Fe^2+^ fluorescence detected by Mito-Ferrogreen (bar = 500 μm). **(B)** TUNEL staining fluorescence intensity (*n* = 15). **(C)** Mitochondrial Fe^2+^ fluorescence intensity (*n* = 15). Results are expressed as the mean ± S.D. ^*^ *P* < 0.05, ^**^ *P* < 0.01 vs the MOD group; ^#^ *P* < 0.05, ^##^ *P* < 0.01. vs the FA group.

Tunel and DAPI double-staining revealed that fluorescence intensity significantly increased in the MOD, FG-4592, and FA + FG-4592 groups compared to the CTRL group (*P* < 0.01). In contrast, FA, Oltipraz, and FA + Oltipraz treatments markedly reduced fluorescence intensity compared to the MOD group (*P* < 0.01), with FG-4592 antagonizing FA’s effect (*P* < 0.01) (Figure 4A, B). These results indicate that FA suppresses ox-LDL-induced apoptosis in RAW 264.7 cells by inhibiting the HIF-1 signaling pathway.

Similarly, Mito-ferrogreen assays showed elevated mitochondrial Fe^2+^ levels in the MOD, FG-4592, and FA + FG-4592 groups compared to the CTRL group (*P* < 0.01), while FA, Oltipraz, and FA + Oltipraz treatments significantly reduced Fe^2+^ content (*P* < 0.01). FG-4592 again antagonized FA’s effect (*P* < 0.01) (Figure 4A, C), suggesting that FA mitigates ox-LDL-induced ferroptosis by reducing mitochondrial Fe^2+^ accumulation via the HIF-1 signaling pathway.

### 3.5 FA improves ox-LDL-induced expression of ferroptosis and apoptosis-related proteins in RAW 264.7 cells

**FIGURE 5:**
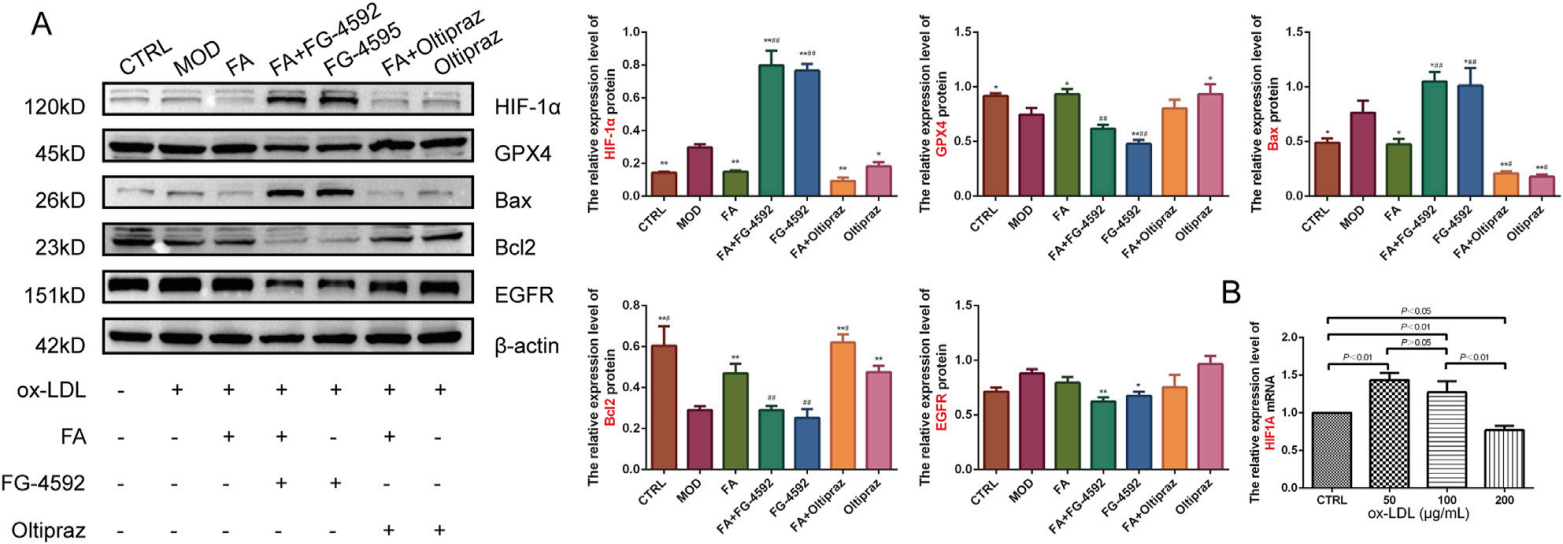
The effects of FA on ox-LDL-induced expression levels of HIF-1α, GPX4, Bax, Bcl2, and EGFR proteins in RAW264.7 cells. **(A)** Relative expression levels of HIF-1α, GPX4, Bax, Bcl2, and EGFR proteins detected by Western blot (*n* = 3). **(B)** Relative expression levels of the HIF1A gene detected by qRT-PCR in RAW 264.7 cells treated with different doses of ox-LDL (*n* = 6). Results are expressed as the mean ± S.D. ^*^ *P* < 0.05, ^**^ *P* < 0.01 vs the MOD group; ^#^ *P* < 0.05, ^##^ *P* < 0.01. vs the FA group.

To further verify the mechanism of FA on AS, the expression levels of HIF-1α, GPX4, Bcl2, Bax, and EGFR proteins were assessed by Western blot. Compared to the CTRL group, the MOD group upregulated HIF-1α (*P* < 0.01) and Bax (*P* < 0.01) expression and downregulated GPX4 (*P* < 0.05) and Bcl2 (*P* < 0.01) expression. FA and Oltipraz treatments reversed these changes (*P* < 0.05), whereas FG-4592 antagonized the effects of FA (*P* < 0.01) (Figure 5A). These results indicate that FA modulates the expression of GPX4, Bcl2, and Bax by inhibiting the HIF-1 signaling pathway.

To further explore the role of HIF-1 signaling, HIF1A gene expression under different ox-LDL concentrations was analyzed. HIF1A gene expression was significantly upregulated in the 50 (*P* < 0.01) and 100 (*P* < 0.01) μg/mL ox-LDL groups compared to the CTRL group, but downregulated in the 200 μg/mL ox-LDL group (*P* < 0.05). HIF1A expression showed a significant decrease with increasing ox-LDL concentrations (Figure 5B).

## 4 Discussion

Network analyses suggest that FA may treat AS through the HIF-1 signaling pathway. Ferroptosis and apoptosis-related genes are similarly enriched in the HIF-1 signaling pathway, indicating a potential link between these processes and AS. HIF-1α, the most important regulator of the hypoxic response, has been shown to affect the pathogenesis of AS through various molecular and cellular events (Jain et al., 2018). Abnormal lipid metabolism increases the synthesis and decreases the breakdown of lipids, leading to excessive lipid deposition in the blood vessel wall. This deposition narrows the lumen of the blood vessel, reducing blood flow and oxygen-carrying capacity. HIF-1α, a sensitive factor for hypoxia, is activated under these conditions, suggesting that the HIF-1 signaling pathway may be a potential mechanism for AS development. This study hypothesized that lipid deposition in the blood vessel wall, vascular stiffening, and plaque formation narrow the lumen of the vessel, reducing blood flow and oxygen-carrying capacity. This causes hypoxia in the body, impairs mitochondrial function, and ultimately affects the development of AS. Mitochondria are also major producers of ROS, and mitochondrial damage increases ROS production. The accumulation of ROS may trigger the development of apoptosis and ferroptosis. Therefore, a pathway of dyslipidemia-hypoxia-HIF-1 signaling pathway-mitochondria-ROS-apoptosis and ferroptosis-AS has been proposed to elucidate the potential mechanisms of FA for the treatment of AS. Among these, dyslipidemia caused by abnormal lipid metabolism may be the initial step in the progression of AS.

Previous studies found that DiDang decoction improves lipid metabolism by activating the HIF-1 signaling pathway (Wu et al., 2023). Based on these findings, it was hypothesized that FA might also act by activating the HIF-1 signaling pathway. However, unexpectedly, WB and qRT-PCR experiments showed that FA instead inhibited the HIF-1 signaling pathway, which was inconsistent with previous speculation. One reason for this discrepancy may be the difference in cell lines. Previous studies used free fatty acids to induce abnormal hepatic lipid metabolism in human normal L02 hepatocytes, whereas this study used ox-LDL-induced RAW 264.7 cells to construct a foam cell model. The liver is the main organ of lipid metabolism, and activating HIF-1α in hepatocytes effectively improves hepatic lipid metabolism and hypoxia, thereby delaying the development of AS. HIF-1α exhibits a protective effect in the liver by inhibiting hepatic lipid accumulation and a destructive effect by promoting hepatic fibrosis (Chu et al., 2022). Early high-fat diets upregulate HIF-1α expression in the liver, improving hepatic lipid metabolism and indirectly delaying AS, which was confirmed by many experiments showing that hepatocyte-specific defects in HIF-1α led to more severe hepatocellular steatosis and lipid accumulation (He et al., 2021; Arai et al., 2018; Nishiyama et al., 2012; Yoo et al., 2014), but promoted liver fibrosis development (Han et al., 2019; Mesarwi et al., 2016). HIF-1α also exhibits some protective effects in early AS, but prolonged activation of HIF-1α induces endothelial cell dysfunction, proliferation, angiogenesis, and inflammation; promotes smooth muscle cell proliferation and migration; and affects macrophage function and lipid efflux, thereby contributing to the development of AS (Jain et al., 2018; Liu et al., 2020). From the perspective of macrophage lipid metabolism, inhibition of HIF-1α inhibits lipid uptake by macrophages and improves hypoxia, which in turn delays the progression of AS.

Additionally, HIF-1α activity is downregulated with increasing lipid infiltration and persistent hypoxia, which may contribute to the observed discrepancy. This study also found that HIF1A expression was downregulated with increasing ox-LDL concentration. Yukino Kobayashi explained that an increase in ROS could be one of the reasons (Kobayashi et al., 2021). Prolonged hypoxia or increased ROS levels induce Ref-1, which activates HIF-1α transcriptional activity. However, the activation of HIF-1α via Ref-1 also induces PHD2 and FIH-1, leading to HIF-1α feedback regulation (Kobayashi et al., 2021). Moreover, HIF-1α may promote abnormal lipid metabolism by shifting cellular energy production from aerobic oxidation to anaerobic glycolysis, causing an imbalance in energy metabolism and leading to downregulation of HIF-1α (Thomas and Ashcroft, 2019). Thus, HIF-1α is usually upregulated in the early stages of high-fat diets, compensating to some extent for improved lipid metabolism. However, persistent activation of HIF-1α promotes processes such as inflammation, endothelial damage, and angiogenesis, which accelerate the development of AS. In response, the body downregulate HIF-1α expression in persistent high-fat and hypoxic environments, but this is not sufficient to restore the pathologic changes. The first part of this study showed that FA effectively improved lipid metabolism in ox-LDL-induced RAW cells, while FG-4592 antagonized the lipid-lowering effect of FA, suggesting that FA may inhibit the HIF-1 signaling pathway to improve lipid metabolism.

Mitochondria are the main organelles for oxygen consumption, and their redox function plays a decisive role in the development of AS. Enhancing mitochondrial oxidative metabolism can promote fatty acid degradation, reduce intracellular lipid accumulation, limit foam cell formation, and delay the onset of AS (Oliveira and Vercesi, 2020; Wei et al., 2018). During hypoxia, organisms adapt by changing the morphology and function of mitochondria through compensatory mechanisms, which affect energy metabolism and contribute to the formation of AS. When cells are hypoxic, mitochondrial ROS are overproduced. The increased ROS prevent the hydroxylation of HIF-1α, which induces HIF-1 protein synthesis, promotes mitophagy, and avoids the continued production of ROS (Klimova and Chandel, 2008; Semenza, 2011; Yang et al., 2019; Zhang et al., 2008). Moreover, HIF-1α can directly target mitochondria, reducing ROS production and preventing oxidative stress-induced apoptosis (Li et al., 2019). It is evident that HIF-1 regulates the “mitochondrial biosynthesis process,” controlling the renewal, content, and quantity of mitochondria, thus limiting mitochondrial ROS production and enhancing the body’s tolerance to hypoxic damage (Thomas and Ashcroft, 2019).The second part of the experiment confirmed that FA can improve mitochondrial function and reduce ROS content. However, FG-4592 antagonized the effect of FA, indicating that FA reduces mitochondrial damage and ROS production by inhibiting the HIF-1 signaling pathway.

The role of mitochondria in apoptosis is definitive, and they are even considered the executioners of apoptosis. The continuous accumulation of ROS leads to the persistent opening of mitochondrial pores, loss of membrane potential, and disruption of the mitochondrial respiratory chain, ultimately promoting apoptosis (Bauer and Murphy, 2020; Zorov et al., 2014). However, the relationship between mitochondria and ferroptosis has not been fully clarified, and many controversies remain. The third part of this study demonstrated that FA can improve mitochondrial function, thereby inhibiting apoptosis and reducing mitochondrial Fe^2+^ content, thus suppressing ferroptosis. These findings confirm the relationship between mitochondrial dysfunction and ferroptosis, showing that mitochondrial damage promotes ferroptosis. One potential mechanism is that mitochondria may inhibit ferroptosis by expressing mitochondrial ferritin, a protein that promotes iron storage within mitochondria, thereby reducing free iron levels and preventing iron-mediated lipid peroxidation (Fang et al., 2019). Additionally, mitochondrial damage generates excess ROS, which further promotes ferroptosis by enhancing lipid peroxidation-a key driver of ferroptotic cell death (Wu et al., 2023).

Further experiments revealed that FG-4592 antagonized the anti-apoptotic and anti-ferroptosis effects of FA. This suggests that FA may improve mitochondrial function and inhibit ferroptosis and apoptosis through the inhibition of the HIF-1 signaling pathway. HIF-1 is indirectly involved in apoptosis by regulating the transcription of genes encoding pro-apoptotic (e.g., Bax) or anti-apoptotic (e.g., Bcl-2) factors, potentially through transcriptional activation or repression (Fuhrmann and Brüne, 2017). However, the relationship between HIF-1 and ferroptosis remains unclear. Studies have found that HIF-1, regulated by hypoxia, increases iron uptake, which affects ferroptosis sensitivity or resists ferroptosis by promoting the expression of SLC7A11, a key component of the cystine/glutamate antiporter system (Gao et al., 2023). In patients with AS, elevated HIF-1α expression and downregulation of GPX4 expression are commonly observed. In mice fed a high-fat diet, HIF-1α expression increases as AS progresses, resulting in higher levels of oxidative stress. Conversely, downregulation of HIF-1α expression reduces ROS production, increases glutathione levels, inhibits ferroptosis, and thus alleviates AS (Cai and Luo, 2024). This study demonstrated that FA ameliorated ox-LDL-induced ferroptosis and apoptosis in RAW 264.7 cells by inhibiting the HIF-1 signaling pathway.

The development of AS is accompanied by hypoxic conditions, during which HIF-1α is activated. HIF-1α directly induces VEGF, endothelin-1, and MMPs in endothelial cells to promote angiogenesis, thereby improving blood supply and oxygenation of the heart and vascular tissues and enhancing vascular function (Knutson et al., 2021). However, over-activation of the HIF-1 signaling pathway induces vascular smooth muscle cell proliferation by up-regulating factors such as CD98 and macrophage migration inhibitory factor. Additionally, over-activation promotes a pro-inflammatory state and increases apoptosis while inhibiting macrophage lipid metabolism, thereby facilitating foam cell formation, a key event in AS progression. Excessive activation of HIF-1α may lead to undesirable physiological effects, including enhanced cell proliferation, inflammation, and fibrosis (Knutson et al., 2021). Thus, in the early stages of AS, HIF-1α exerts a compensatory protective effect by promoting angiogenesis and improving tissue oxygenation. However, persistent lipid infiltration and chronic hypoxia disrupt lipid metabolism, promote ferroptosis and apoptosis, and ultimately drive AS progression. Therefore, regulating the HIF-1 signaling pathway at the appropriate time is critical for AS treatment. This study demonstrates that FA not only improves lipid metabolism and inhibits apoptosis and ferroptosis but also suppresses the expression of inflammation-related genes. These beneficial effects may be mediated through the inhibition of the HIF-1 signaling pathway.

In conclusion, the network analysis in this study revealed that FA may regulate ferroptosis and apoptosis through the HIF-1 signaling pathway for the treatment of AS. Preliminary qRT-PCR experiments showed that FA regulates the expression of genes related to the HIF-1 signaling pathway, ferroptosis, and apoptosis. Subsequently, a foam cell model was constructed using ox-LDL-induced RAW 264.7 cells for experimental validation. The results showed that FA inhibited foam cell lipid accumulation, enhanced cell viability, improved mitochondrial morphology and function, reduced ROS production, and inhibited ferroptosis and apoptosis, likely through the inhibition of the HIF-1 signaling pathway. This study describes the role of the HIF-1 signaling pathway in lipid metabolism, ferroptosis, and apoptosis, and explores the potential mechanisms of FA on ferroptosis and apoptosis.

Although this study provides valuable insights into the mechanisms of FA in the treatment of AS, many limitations and shortcomings remain. Firstly, network analysis is used as a predictive tool. The selection of databases and results is subjective, and databases are not assessed for false-positive and false-negative results. However, this study screened biological processes and pathways based on the stringent criterion of FDR<0.01 and explored the potential relationship of ferroptosis and apoptosis-related genes with the HIF-1 signaling pathway as a node using enrichment analysis, with qRT-PCR for initial validation. Additionally, FA is a small polyphenol molecule with the potential for false-positive results from network analysis and a large number of potential false-positive results from molecular docking with many proteins. However, molecular docking was used in this study as a preliminary exploration, and the results were validated experimentally. Moreover, the results of this study were only validated in RAW 264.7 cells. Further studies are still needed to verify the validity of these findings in other cell types (e.g., endothelial cells, smooth muscle cells) and animal models. Finally, the potential involvement of other signaling pathways (e.g., NF-kB, Nrf2, AMPK) in the effects of FA and their interactions with the HIF-1 signaling pathway, as well as the deeper mechanisms of the HIF-1 signaling pathway, should be investigated.

## 5 Conclusion

FA improves mitochondrial function and suppresses ferroptosis and apoptosis by inhibiting the HIF-1 signaling pathway, thereby treating AS.

## Data Availability

The original contributions presented in the study are included in the article/Supplementary Material, further inquiries can be directed to the corresponding authors.

## Abbreviations

AS, Atherosclerosis; DMSO, Dimethyl Sulfoxide; FA, Ferulic Acid; ox-LDL, Oxidized Low-Density Lipoprotein; ORO, Oil Red O, PBS, Phosphate-Buffered Saline; qRT-PCR, Quantitative Real-Time Polymerase Chain Reaction; ROS, Reactive Oxygen Species; TEM, Transmission Electron Microscope.
