# Supplementary figures and images for: Ferulic acid inhibits ox-LDL-induced ferroptosis and apoptosis in RAW 264.7 cells via the HIF-1 signaling pathway
Source: Front Pharmacol. 2025 Mar 18;16:1524736. doi: 10.3389/fphar.2025.1524736 (PMC11958962; doi:10.3389/fphar.2025.1524736)

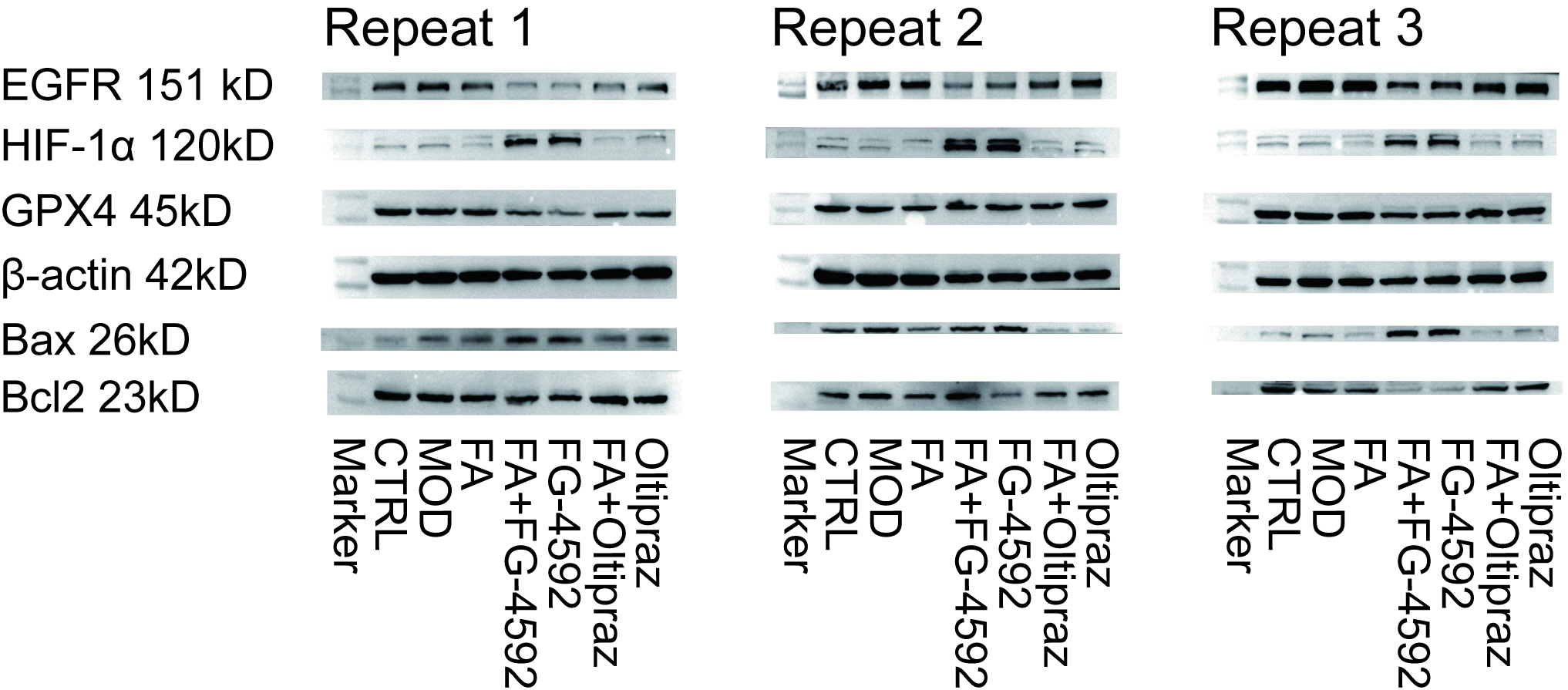

Supplement: Supplementary file 1 [file Image1.tif]
